# Case of a Negro Woman, Who Gave Birth to Twins of Different Color

**Published:** 1849-11

**Authors:** 


*Case of a Negro woman, who gave birth to twins of different color.* B. R. Carter, M.D. Virginia. (Communicated in a letter to one of the Editors.) I promised you when I left Philadelphia, that on reaching home, I would

---

try and find out something concerning the woman who had twins, the children being unlike in color. The following is what I have ascertained in regard to the case. The negro woman Winny, is twenty-three years old, of good constitution, and *as black as the ace of spades*. She has borne three children previously to this labor. She states that in the month of April, 1848, she had connection with a white man, and on the day following, with a black one. Some week or ten days elapsed, when her catameniæ failed to appear. After this she had the ordinary symptoms of pregnancy, the nausea and vomiting being more distressing than in her previous pregnancies. In February, 1849, about the middle of the month, she was delivered of twins. The dark colored child was first delivered and afterwards the mulatto. The children were robust at birth. One of them is a mulatto, and the other is as dark as negro children generally are. The woman is certain that they were begotten by different fathers, and this is he conclusion to which all have come who have seen the children.—*Medical Examiner.*

